# Seeking Clearer Recommendations for Hand Hygiene in Communities Facing Ebola: A Randomized Trial Investigating the Impact of Six Handwashing Methods on Skin Irritation and Dermatitis

**DOI:** 10.1371/journal.pone.0167378

**Published:** 2016-12-28

**Authors:** Marlene K. Wolfe, Emma Wells, Brittany Mitro, Anne Marie Desmarais, Pamela Scheinman, Daniele Lantagne

**Affiliations:** 1 Department of Civil and Environmental Engineering, Tuft University, Medford, Massachusetts, United States of America; 2 Department of Dermatology, Brigham and Women’s Hospital, Boston, Massachusetts, United States of America; University of Ottawa, CANADA

## Abstract

To prevent disease transmission, 0.05% chlorine solution is commonly recommended for handwashing in Ebola Treatment Units. In the 2014 West Africa outbreak this recommendation was widely extended to community settings, although many organizations recommend soap and hand sanitizer over chlorine. To evaluate skin irritation caused by frequent handwashing that may increase transmission risk in Ebola-affected communities, we conducted a randomized trial with 91 subjects who washed their hands 10 times a day for 28 days. Subjects used soap and water, sanitizer, or one of four chlorine solutions used by Ebola responders (calcium hypochlorite (HTH), sodium dichloroisocyanurate (NaDCC), and generated or pH-stabilized sodium hypochlorite (NaOCl)). Outcomes were self-reported hand feel, irritation as measured by the Hand Eczema Score Index (HECSI) (range 0–360), signs of transmission risk (e.g., cracking), and dermatitis diagnosis. All groups experienced statistically significant increases in HECSI score. Subjects using sanitizer had the smallest increases, followed by higher pH chlorine solutions (HTH and stabilized NaOCl), and soap and water. The greatest increases were among neutral pH chlorine solutions (NaDCC and generated NaOCl). Signs of irritation related to higher transmission risk were observed most frequently in subjects using soap and least frequently by those using sanitizer or HTH. Despite these irritation increases, all methods represented minor changes in HECSI score. Average HECSI score was only 9.10 at endline (range 1–33) and 4% (4/91) of subjects were diagnosed with dermatitis, one each in four groups. Each handwashing method has benefits and drawbacks: soap is widely available and inexpensive, but requires water and does not inactivate the virus; sanitizer is easy-to use and effective but expensive and unacceptable to many communities, and chlorine is easy-to-use but difficult to produce properly and distribute. Overall, we recommend Ebola responders and communities use whichever handwashing method(s) are most acceptable, available, and sustainable for community handwashing.

**Trial Registration:** International Standard Randomized Controlled Trial Registry ISRCTN89815514

## Introduction

First characterized following an outbreak in Zaire (now the Democratic Republic of Congo) in 1977 [1], Ebola causes severe disease and has a case fatality rate (CFR) ranging from 25–100% [2]. The disease begins abruptly and presents with a high fever, headache, muscle pain, weakness, diarrhea, and vomiting, along with a characteristic rash and hemorrhaging in some patients [3]. The 2014 Ebola Virus Disease (EVD) outbreak in West Africa was the first widespread outbreak and the largest to date. From December 2013 to Jan 2016 there have been 28,638 cases of Ebola and 11,316 deaths, mostly within West Africa but also spreading to ten countries including the United States and several European nations [4].

Prior to the 2014 outbreak, Ebola emerged in remote, low population density settings, and outbreaks were contained primarily by medical responders. The West African outbreak began in Guinea with the death of a 2-year-old child in a remote forested area. It then spread to urban areas via healthcare workers, and then grew exponentially after the death of a businessman in the capital of Conakry [5]. This was the first reported introduction of Ebola into densely populated urban areas. Here the virus continued to persist solely through human-to-human transmission with a dynamic and level of risk not previously seen [6–8].

The Ebola virus is transmitted through contact with an infected person or animal, their bodily fluids, or contaminated surfaces [9,10]. Contact with fluids, needle stick injuries, and skin openings have resulted in more severe cases and higher fatality rates [11]. Ebola patients produce a voluminous amount of infectious fluids (vomit, diarrhea, blood hemorrhage) and the use of personal protective equipment (PPE) and rigorous standards of handwashing are essential in Ebola Treatment Units (ETUs) [12]. Methods of handwashing that disinfect hands while preserving an intact skin barrier are an important defense against highly infectious diseases [2]. The commonly recommended methods of handwashing in Ebola settings utilize soap and water, alcohol based hand sanitizer (ABHS), or 0.05% chlorine solution. While the efficacy of these solutions is important, the safety of these solutions on skin during frequent handwashing is also significant in an Ebola context.

There is a lack of clear evidence on the safety of these three handwashing methods that has led to inconsistent and even contradictory international guidelines. Doctors Without Borders (MSF) recommends that buckets of 0.05% chlorine solution are prepared and placed around ETUs for regular hand washing use by staff and caregivers [13]. By contrast, both the World Health Organization (WHO) and the United States Centers for Disease Control and Prevention (CDC) recommend handwashing with soap and water or hand sanitizer, stating that handwashing with chlorine “is not ideal because this may lead to skin lesions, which could increase risk of infection”, because breaks in the skin facilitate entry of the virus into the bloodstream [14]. CDC and WHO also state that frequent use of any handwashing method may result in disruption of the skin barrier, and recommend that chlorine solution can be used for handwashing only if other options are not available [15,16].

In an effort to contain the sustained transmission of Ebola in urban centers, these handwashing recommendations initially meant for ETU settings were extended to the community level in the West African outbreak. In particular, handwashing with 0.05% chlorine solution has been commonly adopted by government, health, and commercial facilities in West Africa [17]. Responders observed a proliferation of handwashing stations and increased interest in using chlorine across outbreak-affected areas; fact sheets were developed to guide response, and grant money was sought to expand chlorine use and testing, but there was uncertainty about best practices and it is unclear how many of these projects were completed.

All of the recommended handwashing methods can lead to dermatitis, a condition of skin inflammation and skin barrier disruption that can cause transepidermal water loss, itching, redness, swelling, and an increase in disease transmission risk [18]. There are two types of dermatitis: allergic contact dermatitis (ACD) and irritant contact dermatitis (ICD). ACD is a specific allergic response (Type IV delayed hypersensitivity reaction) to a particular substance (antigen) in previously sensitized individuals [19]. The gold standard for diagnosing ACD is by performing a type of topical skin testing called patch testing. ICD, in contrast, does not require specific sensitization and can be caused by substances that are strongly irritating to many people [20]. Chemicals such as sulfuric acid or hydrochloric acid can cause acute ICD. Materials such as soap and water can lead to chronic ICD as they repetitively cause dehydration of the stratum corneum, the lipid- rich, outer protective layer of the skin [21]. This disrupts the skin barrier and causes transepidermal water loss with resultant inflammation.

Because ICD does not require specific sensitization and can occur in a large percentage of the population, it presents a worrisome risk for groups required to perform numerous handwashings with potentially irritating solutions and therefore put individuals at risk of Ebola infection. ICD has been demonstrated in users of soap and water [16,22], ABHS [23,24], and chlorine solution [25–28]. However, this assessment of the impact of chlorine solutions on skin relies heavily on observational studies and case reports of accidental exposure to concentrations much higher than those recommended for handwashing in Ebola contexts [16,22,26,29–32]. The compounds commonly used to provide free chlorine in solution for disinfection in emergencies—sodium hypochlorite (NaOCl), calcium hypochlorite (HTH), and sodium dichloroisocynaurate (NaDCC)–also differ in their chemical properties and may differ in potential to cause ICD. Lastly, known effect modifiers and confounding factors (including weather and baseline reactivity) are often not considered in these observational studies and case reports [18].

Differences in recommendations cause us to question the best methods to prevent disease transmission when ETU recommendations are extended to the community setting. We investigated the impact of six recommended handwashing methods on skin irritation and dermatitis to better understand the risk that community level handwashing may pose during an Ebola outbreak.

## Methods

We conducted a randomized study during which subjects washed their hands 10 times per day for 28 days, and other potentially irritating personal-care products and activities were controlled. The six handwashing methods were soap and water, ABHS, 0.05% HTH solution, 0.05% NaDCC solution, 0.05% NaOCl solution produced with an electrochlorinator, and stabilized 0.05% NaOCl solution produced from laboratory grade NaOCl stock.

The study took place on the Tufts University campus in Medford, MA beginning with recruitment on September 11, 2015 and concluding with endline data collection on November 18, 2015. The clinical outcomes were: 1) patch test results, 2) self-rated hand evaluation, (secondary outcomes) 3) Hand Eczema Score Index (HECSI) evaluation, and 4) diagnosis of dermatitis (primary outcomes). We completed the following activities during which these factors were assessed: 1) recruitment and enrollment, 2) baseline assessment and patch testing, 3) daily handwashing intervention and evaluation, and 4) endline patch testing. The study was approved by the Institutional Review Board at Tufts Medical Center and Tufts University Health Sciences Campus (#11818); Harvard University ceded review to the Tufts Institutional Review Board. Here we describe first the key study outcomes, and then the study activities during which outcomes were assessed. This study was not registered as a clinical trial prior to the start of subject enrollment because the Institutional Review Board did not consider the study to be a clinical trial. The authors confirm that all ongoing and related trials for these interventions are registered.

### Outcome Assessment

#### Outcome #1—Allergy Testing

Patch testing is the gold standard for assessing whether a substance induces an allergic reaction on the skin, and testing was performed prior to the intervention and after the completion of the study period according to a typical patch testing protocol used by dermatologists [33]. Researchers were trained to apply the patch test materials by a board-certified dermatologist. We added ~17 μL of each of the six study handwashing substances to filter paper placed in Finn Chambers on Scanpor tape (SmartPractice, Phoenix, AZ). The 0.05% chlorine solutions and hand sanitizer were used without modification, and bar soap was emulsified in distilled water (1 gram in 10 mL) and a 10:1 dilution of the resulting mixture prepared (1% dilution). After substances were added, excess liquid was dabbed away using separate cotton swabs and the tape was applied to the subject’s upper back. Subjects were asked to wear the patch without disturbing it for 48hrs, and then to remove it. Upon removal, subjects outlined the patch area using a provided surgical marker, and submitted self-administered photos of the testing area through an online form 48 and 96 hours post-application for evaluation. On the seventh day after application, the patch area was visually examined by a researcher to confirm test results. The researcher also submitted day 7 photos for evaluation by the board-certified dermatologist.

#### Outcome #2—Self-Rated Hand Score

Subjects used a scale from 0–10 developed by the research team to self-rate the level of discomfort that they were currently experiencing on their hands along with nine different symptoms, including itchiness, pain, redness, flaking, cracks in the skin, skin thickening, swelling, bumps, and blisters.

#### Outcome #3—Hand Eczema Severity Index Score

The HECSI system is a sensitive and standardized clinical grading approach for hand eczema and dermatitis, and allows comparison of minor changes in skin irritation [34]. The system includes six irritation signs (erythema/redness, papulation/bumps of skin, vesicles/blisters, fissuring/cracking, scaling/flaking, and edema/swelling) and their severity (0, none present; 1, mild disease; 2, moderate disease; 3, severe disease). The score also incorporates the surface area covered by any sign of irritation by splitting hands into 5 areas (fingertips, fingers without tips, palms, backs of hands, and wrists) and asking the scorer to describe the percentage surface covered (0, 0%; 1, 1–25%; 2, 26–50%; 3, 51–75%; 4, 76–100%). These values are used to calculate the final HECSI score, a number ranging from 0–360. Two researchers were trained by a board certified dermatologist to recognize the signs of dermatitis assessed using the HECSI score, and participated in several practice sessions prior to data collection. During data collection, HECSI scores were assessed by a single researcher viewing subjects’ hands in person. Researchers were partially blinded to intervention group—it was occasionally possible for researchers to infer group assignment from sights and smells as they were assessing subjects in person, but they were not informed of the treatment for each participant and made every effort to avoid this information.

Subjects would receive a consultation with the dermatologist if they received a HECSI score >68. To ensure protection of subjects, two photos, one of the backs of hand and one of the palms were also taken daily, uploaded to a secure study server nightly, and reviewed within 24 hours by the board-certified dermatologist to screen for issues of clinical concern. Subjects would be removed from the study if they received a HECSI score >270, or if concern was noted by the dermatologist.

#### Outcome #4—Dermatitis

Dermatitis was diagnosed by a board-certified dermatologist who examined subjects in person on campus at Tufts University on the last day of the study and classified cases as mild, moderate, or severe. The dermatologist was blinded to the intervention arm of each subject examined.

### Study Activities

#### Enrollment

**Fig 1:**
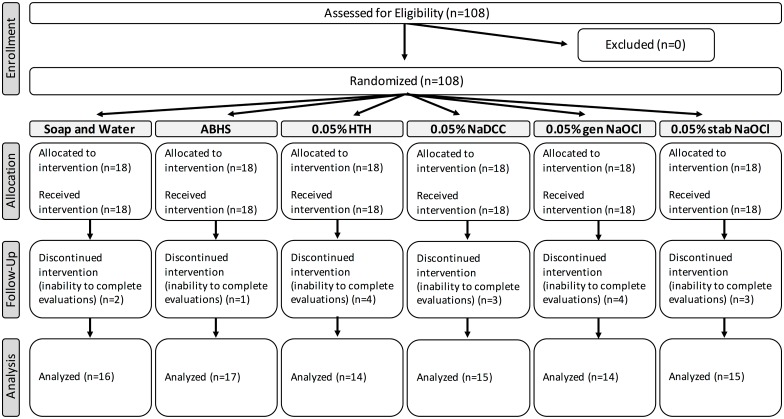
Summary of Trial Design.

A total of 108 subjects were recruited and signed written informed consent forms (Fig 1). Subjects were required to be age 18 to 65 with no history of dermatitis or baseline skin abnormalities, allergy to any of the handwashing materials, or mental health problems related to hygiene practices. Because studies on community handwashing and dermatitis were not available to establish sample size and because the study was very resource intensive, the investigators and IRB were unwilling to subject more than the minimum number of subjects to even a minimal risk preliminary study. The size of the study was chosen to allow for a commonly agreed-upon minimum of 15 for statistical tests of comparison between groups and increased slightly to 18 per group to allow for attrition. Subjects were also excluded if they were pregnant or trying to become pregnant, or were currently working in an industry that exposed them to frequent handwashing (including, but not limited to, healthcare workers, food service, and salon workers).

#### Baseline Assessment

One week before the intervention, a baseline assessment was used to assess usual hygiene behaviors and confirm that subjects met study criteria. Researchers administered a short survey, including questions about demographics; history of atopic (genetic allergic) disposition; handwashing behavior; and current skin, household, and hygiene product use. Additionally, enumerators asked subjects to respond to the self-rated hand score assessment. Subjects’ hands were examined and scored on the HECSI scale by a trained researcher. Lastly, subjects were patch tested.

#### Intervention

Subjects who completed the baseline assessment and met all study criteria were randomly assigned to one of six handwashing treatment arms (soap and water, ABHS, HTH, NaDCC, generated NaOCl or stabilized NaOCl). Block randomization with a block size of 18 was performed by a researcher not involved in day-to-day study activities after all subjects were enrolled using a random number generator to assign 18 subjects per arm. Subjects were informed of whether they were part of the soap and water, hand sanitizer, or chlorine arms and given a drawstring bag with materials and instructions for handwashing. Subjects were asked to wash their hands ten times each day with their assigned method according to the instructions provided, and returned to the study office each day for 28 days for hand examination, supply refill, and to answer a short questionnaire.

Soap and water and ABHS handwashing protocols were based on WHO’s hand hygiene technique guidelines [35], and chlorine protocols were based on MSF guidelines [13]. Subjects in the soap and water arm were provided with Dove white beauty bar soap (Unilever, Trumbull, CT) with a carrying case. This soap was chosen as a realistic approximation of soaps for field use. They were asked to rub their lathered hands together for at least 20 seconds, taking care to scrub all parts of the hands, rinse in running water and dry hands with a clean towel. Subjects in the ABHS arm were provided with Purell Advanced Instant Hand Sanitizer with 70% Ethyl Alcohol (GOJO Industries, Inc. Akron, OH) in a small generic squeeze bottle and were asked to dispense an amount able to coat the hands completely and rub over all surfaces until dry. Subjects in each of the chlorine arms were provided with a 1-liter opaque amber HDPE bottle and a measuring cup. They were asked to wash their hands by pouring 100mL of solution onto both hands and rubbing together until dry, and not rinse hands with water after washing.

Subjects were asked to refrain from using their usual shampoo, conditioner, soap, and moisturizer and to avoid contact with cleaning substances to control for other potential sources of irritation. Subjects were provided with donated Free and Clear Shampoo and Conditioner and a Vanicream^™^ Cleansing Bar (Pharmaceutical Specialties, Inc. Rochester, MN) for use when showering or for handwashing beyond 10 times a day, and commercially available heavy-duty vinyl gloves (Allerderm, Phoenix, AZ) for use during household activities such as dishwashing. Subjects were also provided with commercially available Vaseline petroleum jelly (Unilever, Trumbull, CT) as a moisturizer. These products were chosen because they are hypoallergenic and fragrance free. In particular, control soap was chosen to be less irritating than intervention soap to prevent interference with intervention effects.

#### Chlorine Solution Preparation

Chlorine solutions were produced daily in the Environmental Sustainability Laboratory at Tufts University and their concentrations confirmed to be within 10% of a target 0.05% solution daily using Hach Iodometric Titration Method 8209 (Hach Company, Loveland, CO). NaDCC solution was produced using donated Klorsept granules (previously Aquatabs) with 50% active chlorine (Medentech, Wexford, Ireland). HTH was produced from commercially available granular calcium hypochlorite with 65% available chlorine (Acros Organics, New Jersey, USA), and the solution allowed to settle for 24 hours and decanted to avoid precipitate. Two types of NaOCl were produced by: 1) using table salt without added iodine and an Aquachlor on-site sodium hypochlorite generator (International Equipment & Systems, Inc. Miami, FL), and 2) diluting a 5.25% lab-grade pH stabilized bleach stock solution (Valtech, Zellenople, PA). Milli-Q deionized water was used for mixing all solutions, and pH was measured daily using Hanna Instruments HI 9811–5 portable pH/EC/TDS/°C meter, calibrated with non-expired solutions (Hanna Instruments, Woonsocket, RI). Expected pH values were 6–7 for NaDCC, 10.4–10.8 for HTH, 10.8–11.4 for stabilized NaOCl, and 9 for generated NaOCl.

#### Daily Evaluation

Each day subjects visited the study office for a survey, hand evaluation, and supply refill. Subjects first filled out a self-administered survey asking them about their handwashing behavior and use of provided control products and products outside the protocol. Additionally, subjects were asked about household cleaning activities during the previous day. Subjects then had their hands examined and scored according to the HECSI system by a trained researcher. Lastly, subjects were required to bring their handwashing materials with them each day and were provided with refills as needed. Researchers observed whether or not the materials appeared to be used, recording the amount of chlorine and ABHS remaining in the bottle, and observing if the soap was wet.

#### Endline

On day 28 of the intervention, subjects performed normal daily evaluation activities, and also: 1) reported their level of compliance over the course of the study anonymously on paper forms, identifying themselves only by group assignment, 2) were examined by a board-certified dermatologist for dermatitis, and 3) had a second round of patch testing applied.

### Analysis

Data were collected electronically using Qualtrics software (Qualtrics, Provo, UT), and analysis was completed using Stata 14 (StataCorp LP, College Station, TX). A chi-square test or Fisher’s exact test was used to assess differences in baseline group characteristics. Student’s paired t-test was used to evaluate differences in self-rated hand score and HECSI score from baseline to endline. Linear regression was used to evaluate the association between the HECSI score, intervention arm, and other potential effect modifiers including gender, atopic disposition, weather, and moisturizer use. We constructed two models: 1) using soap as a comparison because it is the most common recommendation, and 2) using the handwashing method with the lowest HECSI score as a comparison. Subjects who reported a history of asthma, seasonal allergies, hayfever, or childhood eczema were categorized as having an atopic disposition. We also divided the six components of the HECSI score into higher and lower transmission risk categories. Higher transmission components included vesicles, scaling, and fissuring (i.e those manifestations with greatest disruption to the skin barrier), and erythema, papulation, and edema were considered lower transmission risk. Logistic regression was used to evaluate the association between the presence/absence of high transmission risk components with the same explanatory variables as the linear regression. There were no adjustments made for multiple comparisons and nominal p-values are reported.

## Results

Overall, 108 people registered for the study, 99 completed the baseline assessment, and 91 completed all study activities. Subjects who left the study prior to the baseline assessment were found not to meet study criteria or removed themselves due to inability to complete all study evaluations. All 8 subjects who left the study after baseline described their reason as an inability to complete daily evaluations with the study team. Thus, all data is presented with an n = 91.

Subjects were 58% female, 57% self-described their race as “white,” and 49% of subjects were classified as having an atopic disposition (Table 1). Broken down further, 41% of subjects reported a history of seasonal allergies, 12% reported asthma, 7% reported childhood eczema, and 5% reported hayfever. There were no significant differences between intervention group breakdown by gender, race, or atopic disposition. In the baseline survey, subjects self-reported that they washed their hands an average of 6.8 times a day (range 1–20), and reported that on the previous day they had used soap an average of 6.5 times (range 1–20) and hand sanitizer an average of 0.9 times (range 0–10). All groups with the exception of NaDCC (p = 0.139) reported a significantly lower level of handwashing at baseline compared to handwashing during the study period (p<0.05). No subject had a positive patch test reaction that was discernable by researchers at baseline or endline, however other skin conditions such as acne or washing away of marks made on skin during testing made it occasionally difficult to view results.

**Table 1:** Descriptive and Compliance Data by Intervention Group.

	Soap (n = 16)	ABHS (n = 17)	HTH (n = 14)	NaDCC (n = 15)	NaOCl (gen) (n = 14)	NaOCl (stab) (n = 15)	Total (n = 91)	Chi square* P-Value
**Race—Black**	6% (1)	6% (1)	0% (0)	0% (0)	7% (1)	7% (1)	4% (4)	0.613
**Race—White**	44% (7)	65% (11)	57% (8)	80% (12)	64% (9)	33% (5)	57% (52)	
**Race—Asian Descent**	31% (5)	24% (4)	29% (4)	7% (1)	14% (2)	40% (6)	24% (22)	
**Race—Multiple**	19% (3)	6% (1)	14% (2)	13% (2)	7% (1)	13% (2)	12% (11)	
**Race—Unknown**	0% (0)	0% (0)	0% (0)	0% (0)	7% (1)	7% (1)	2% (2)	
**Gender (% Male)**	50% (8)	29% (5)	36% (5)	47% (7)	57% (8)	33% (5)	42% (38)	0.60
**Atopic Disposition**	56% (9)	47% (8)	38% (5)	67% (10)	43% (6)	47% (7)	50% (45)	0.63
**Compliance (# handwashes/day)**	8.8	9.2	8.9	9.2	9.3	9.2	9.1	0.90

*Fisher’s exact test used in place of chi-square for variables with frequency<5 in any cell, one-way analysis of variance (ANOVA) calculated comparing mean handwashing among groups for compliance

Subjects self-reported handwashing an average of 8.8 to 9.2 times by study group with their handwashing study method and there was no significant difference in compliance by group found by linear regression (Table 1). All chlorine solution concentrations and pH measurements met quality control requirements and matched expectations (Table 2).

**Table 2:** Average Concentration and pH of Chlorine Solutions.

	HTH	NaDCC	NaOCl (generated)	NaOCl (stabilized)
**Mean Chlorine Concentration**	0.051%	0.051%	0.049%	0.050%
**Mean pH**	10.02	6.55	8.14	9.92

### Self-Reported Irritation

At the baseline assessment, subjects reported an average self-reported hand score of 0.88 out of a possible 90 (range 0–9). On the final intervention day, subjects reported an average score of 1.18 (range 0–11). This change in discomfort over the course of the intervention was not statistically significant (p = 0.262). At baseline and endline the most prevalent self-reported sign of irritation was redness, present in 16% at baseline and 31% at endline.

### HECSI Score

**Fig 2:**
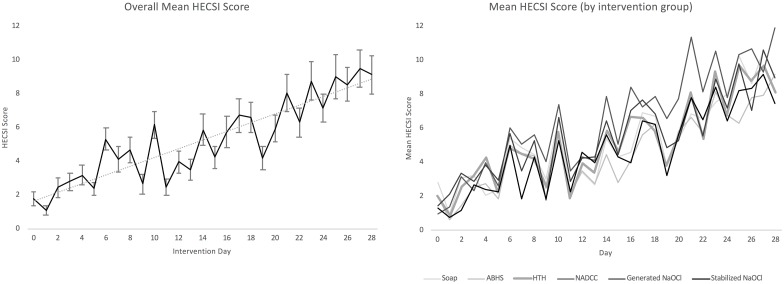
Mean HECSI score overall and by intervention group over time.

At baseline, the average HECSI score was 1.76 out of a possible 360 (range 0–10) (Fig 2). At endline, the average HECSI score was 9.10 (range 1–33). Overall, subjects experienced a higher HECSI score at endline (p<0.001). When stratified by handwashing method, each group also demonstrated a significant difference in HECSI score from baseline to endline (p = 0.002 for soap, p<0.001 for all other groups). Erythema (redness) was the most prevalent sign of irritation observed, present in 53% of subjects at baseline and 100% at endline.

Across all arms, days of handwashing (p<0.001) and male gender (p<0.001) were associated with a higher HECSI score, while atopic disposition (p<0.001) and higher average humidity (p<0.001) were associated with a lower HECSI score (Table 3).

**Table 3:** Multivariate Linear Regression Analysis of HECSI Outcomes.

Variable	*Soap as Reference*			*ABHS as Reference*		
	β	95% CI		β	95% CI	
Days of Handwashing	0.26***	0.24	0.28	0.26***	0.24	0.28
Treatment Type						
* Soap*	--	--	--	0.61*	0.10	1.12
* Alcohol Based Hand Sanitizer*	-0.61*	-1.12	-0.10	--	--	--
* HTH*	-0.12	-0.66	0.41	0.48	-0.04	1.01
* NaDCC*	1.29***	0.77	1.81	1.90***	1.38	2.41
* NaOCl (generated by electrochlorinator)*	0.06	-0.47	0.59	0.67*	0.14	1.20
* NaOCl (from stabilized stock solution)*	-0.46	-0.98	0.06	0.15	-0.36	0.66
Gender	0.82***	0.50	1.14	0.82***	0.50	1.14
Atopic Disposition	-1.09***	-1.40	-0.77	-1.09***	-1.40	-0.77
Average Daily Humidity	-0.05***	-0.06	-0.04	-0.05***	-0.06	-0.04

*p<0.05,

** p<0.01,

***p<0.001,

r^2^ = 0.2642

In comparison to the soap treatment, ABHS resulted in a significantly lower HECSI score (p = 0.019), while NaDCC resulted in a significantly higher HECSI score (p<0.001) (Table 3). We were not able to demonstrate any difference between soap and HTH (p = 0.651), stabilized NaOCl (p = 0.084), or generated NaOCl (p = 0.818). Average daily temperature and use of Vaseline as a moisturizer were originally included in the model, but did not change the results and were removed.

The group with the lowest HECSI was ABHS, which was therefore used as a reference group for a second model. In comparison to the ABHS treatment, soap (p = 0.019), NaDCC (p<0.001), and generated NaOCl (p<0.013) were all associated with a higher HECSI score when corrected for potentially confounding factors and effect modifiers (Table 3). We were not able to demonstrate any difference between ABHS and HTH (p = 0.069) or stabilized NaOCl (p = 0.571). As above, temperature and moisturizer use were removed from the model.

**Fig 3:**
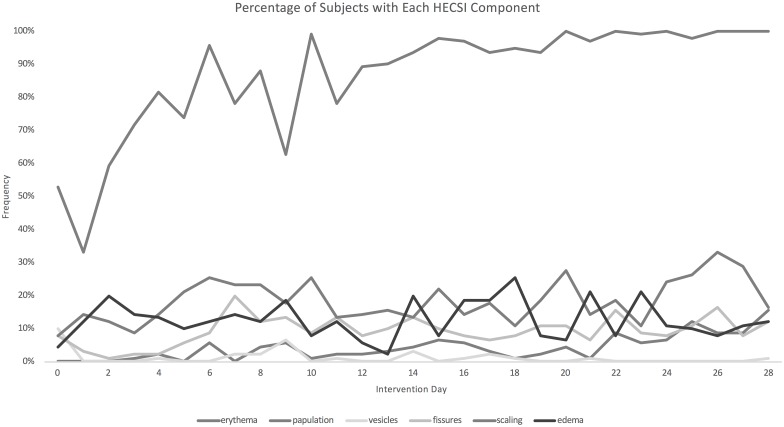
Percentage of subjects displaying each aspect of the HECSI score over time.

Erythema (redness) was the greatest contributor to the HECSI score (Fig 3). Over the course of the study, erythema was observed in 33–100% of subjects, papulation (bumps) in 0–15%, vesicles (blisters) in 0–10%, fissures (cracks) in 1–20%, scaling in 8–29%, and edema (swelling) in 4–25%. Overall, between 11–40% of subjects had some signs of elevated transmission risk over the course of the study.

In logistic regression analysis, days of intervention (p = 0.002) and male gender (p<0.001) were significantly associated with higher odds of transmission risk (Table 4). History of atopic disposition (p<0.001) and Vaseline use (p = 0.019) were both associated with lower odds of transmission risk. Average daily humidity was not significantly associated with transmission risk (p = 0.416).

**Table 4:** Logistic Regression for Signs of Transmission Risk at Endline.

Variables	*Soap as Reference*			*ABHS as Reference*		
	Odds Ratio	95% Conf. Interval		Odds Ratio	95% Conf. Interval	
Days of Handwashing	1.02***	1.01	1.03	1.02***	1.01	1.03
Soap				1.55**	1.13	2.12
ABHS	0.65**	0.47	0.88			
HTH	0.57***	0.41	0.80	0.89	0.63	1.25
NaDCC	0.85	0.62	1.16	1.32	0.95	1.83
NaOCl (generated by electrochlorinator)	0.89	0.66	1.22	1.39	1.00	1.92
NaOCl (from stabilized stock solution)	0.68*	0.50	0.94	1.06	0.76	1.47
Gender	2.72***	2.23	3.33	2.72***	2.23	3.33
Atopic Disposition	0.56***	0.46	0.68	0.56***	0.46	0.68
Vaseline Use	0.89*	0.80	0.98	0.89*	0.80	0.98
Average Daily Humidity	1.00	0.99	1.00	1.00	0.99	1.00

*p<0.05,

** p<0.01,

***p<0.001

In comparison to soap, ABHS (p = 0.006), HTH (p = 0.001), and stabilized NaOCl (p = 0.020) treatments were all associated with a significantly lower odds of transmission risk, and NaDCC (p = 0.306) and generated NaOCl (p = 0.477) did not have significantly different odds (Table 4).

In comparison to ABHS, soap (p = 0.006) and generated NaOCl (p = 0.050) had significantly higher odds of transmission risk, but there was no significant difference with HTH (p = 0.500), NaDCC (0.102), or stabilized NaOCl (p = 0.726) (Table 4).

### Dermatitis Outcomes

At endline, 4% (4/91) subjects presented with clinical dermatitis, one in each of four of the six total groups: soap, NaDCC, generated NaOCl and stabilized NaOCl. There was no significant difference in dermatitis among groups (p = 0.824), and all cases were classified as “mild.”

## Discussion

We completed a randomized study including 91 subjects who washed their hands with six different commonly used methods for 28 days with a high level of compliance. Subjects demonstrated low levels of irritation at baseline, and across all groups there were significant increases in skin irritation. ABHS was associated with the least irritation and fewest signs of potential Ebola transmission risk related to skin barrier disruption followed by high-pH chlorine solutions (HTH and stabilized NaOCl), and then soap and water. Neutral-pH chlorine solutions (NaDCC and generated NaOCl) resulted in the most irritation. Handwashing with soap and water was associated with the greatest odds of signs of potential transmission risk, while ABHS and HTH were associated with lowest odds. These results suggest that irritation varies with handwashing method and chlorine chemistry, particularly pH. Only 4% of subjects developed mild dermatitis by the end of the study period, despite the observed increases in irritation. Thus we found that no handwashing method presented a significant transmission risk by compromising skin integrity.

Our study showed some surprising results, such as the lower odds of transmission risk associated with history of atopic disposition. In many studies atopic diathesis, and specifically atopic dermatitis, has been associated with more severe hand dermatitis [36,37]. Because we excluded subjects with a history of hand dermatitis but did not exclude those with atopic disposition, we postulate our subjects with history of atopy demonstrated adaptive management techniques (such as Vaseline use) to avoid dermatitis. The increased skin irritation and higher odds of signs of transmission risk among men is also surprising as most studies show a higher prevalence of ICD among women. However, this is likely a reflection of impacts of hand hygiene and wet work at home, rather than an actual gender predisposition to dermatitis [37]. Our study subjects (mainly college students in an affluent setting) are less likely to experience differences in activities between genders, or do housework, than the general population. Furthermore, many men in our study commonly performed activities such as lifting weights at the gym, rowing, or breakdancing, which would expose them to friction and other trauma potentially increasing the severity of their hand dermatitis.

The most surprising result of the study was that higher pH dilute chlorine solutions were associated with lower HECSI scores. Both scientific literature and recommendations amongst producers and users of hygiene products suggest that pH-neutral or slightly acidic skin preparations should to help preserve the “acid mantle” of the outer layers of the skin by matching skin pH [21,38,39]. While studies have demonstrated patients with dermatitis have a higher baseline skin pH, and that pH of skin rises for a short time after using basic skin preparations, use of these products has not been linked with a persistent rise in baseline skin pH. Skin appears to buffer this effect quickly, and pH alone has not been shown to be a major contributor to irritation [27,40]. One possible explanation for our results is that handwashing with higher pH solutions may cause a transient, non-irritating rise in skin pH, and that higher-pH solutions interact with epidermal corneocytes in a way which promotes a favorable pH homeostasis over the long term, as we know that the skin actively responds to such challenges [27]. We also postulate that highly reactive hypochlorous acid present in higher concentration in low pH chlorine solutions may impact irritation, although the precise effects of these chlorine byproducts have not been quantified [41].

Other results of our study are consistent with findings in the literature on skin irritation and dermatitis, including the impact of humidity and use of moisturizer as effect modifiers reducing irritation [18]. Our results also raise other important areas of consideration, including: 1) the role of handwashing and irritation compared to other hand damage, 2) the variation within each handwashing method and potential impact on hands, 3) the utility and feasibility of each method in Ebola contexts, and 4) translation of these results to medical personnel.

Maintaining skin integrity is essential to prevent Ebola transmission. We documented a slight increase in skin irritation related to handwashing; however, people often have compromised skin integrity for other reasons. Our subject population of primarily university students had small wounds on their hands such as burns, paper cuts, or blisters from recreational activities. In a population that does more work with their hands, we would expect these percentages to be higher. Thus, community handwashing is unlikely to represent the biggest assault on skin integrity.

We also note that significant variation exists within all three recommended handwashing methods tested. Soap may refer to a range of products, from moisturizing formulas intended for frequent use in hospitals to harsher detergents and bar soaps. Alcohol-based hand rubs produced as gels with elevated levels of glycerine are more hydrating [23]. We expect these differences to have an effect on irritation, and hand hygiene guidelines usually suggest products with humectants to reduce drying [35]. Ebola response guidelines, however, do not emphasize this or note that soaps available in Ebola outbreak areas are often harsher products such as detergents [42,43].

Similarly, chlorine solutions may be made from the range of compounds we tested in this study. Guidelines for chlorine use do not take into account the variation in chlorine properties or specify a recommended type, and these differences have not been well investigated until now. It has been suggested that NaDCC may be preferable because it has a pH similar to that of human skin [39]. However, as discussed, we found that solutions with a lower pH actually resulted in more irritation. Further research should be done on the differences between products within each of the suggested methods, and recommendations should specify the types of hand sanitizer, soap, and chlorine solutions that are least irritating.

Aside from skin irritation, there are benefits and drawbacks to each of the methods. Soap is widely available, familiar, and acceptable, but it requires water, can be easily stolen from handwashing stations, and does not inactivate the Ebola virus. ABHS is the easiest and fastest method, requires no water, and inactivates viruses similar to Ebola. However it is very expensive because it must be imported or produced locally using ingredients which must be imported [44], and it is sometimes prohibited by Muslim communities in West Africa because use can be considered consumption of alcohol [45]. Chlorine solutions are widely accepted for use in Ebola contexts and they are quick methods that inactivate viruses similar to Ebola and do not require water. Unfortunately, producing any chlorine solution requires accurate measuring instruments and access to clean water without chlorine demand. Furthermore, different types of chlorine present additional challenges. The shelf-life of chlorine solutions should be considered (high-pH solutions are noteworthy for having longer shelf-lives than low-pH solutions), and it is essential to choose testing methods that allow responders in the field to confirm that solutions are at the appropriate concentration. HTH and NaDCC are produced from powders that are easy to ship and store, however the HTH solution has a precipitate that can clog pipes. We also found that HTH concentration fluctuated erratically and the solution required adjustment before use. Stock NaOCl solution is purchased as a liquid and can be difficult to transport, and generating NaOCl with an electrochlorinator requires clean water and salt. For both these methods, stock solution must be measured to mix a handwashing solution at the appropriate concentration. These benefits and drawbacks of each method have significant role in determining which methods are used in an Ebola setting.

Our study focused on simulating a level of handwashing typical at the community level during an Ebola outbreak. Healthcare workers wash their hands more frequently than the ten times daily that was required of our subjects—around 2–15 times per hour or 5–42 times per day/shift [35]. More frequent handwashing is likely to lead to more irritation. Healthcare workers in Ebola outbreaks are also required to wear extensive PPE, which is a potential independent source of irritation [46]. It is also possible that occlusion of handwashing substances against the skin surface by PPE after handwashing could lead to increased irritation. While this means that our results are most relevant for community-level handwashing, they also inform further research for handwashing in medical settings both in Ebola contexts and wherever health-care workers commonly report dermatitis and skin irritation from frequent handwashing [32,47].

Our study is limited by differences in context between the Tufts campus in Medford, MA and the centers of the Ebola outbreak in West Africa, including weather and subject characteristics, and also by sample size. The weather during our investigation was cooler and drier than most Ebola affected areas, and we did not collect information on time spent outdoors. The majority of our subjects were both young and full time students. Skin loses collagen and elasticity as people age [48,49], and results might vary in a population that spends more time working with their hands. Our subjects were mostly Caucasian or of Asian descent, and there is some evidence that there are differences in skin physiology by race or pigmentation. Some studies suggest that individuals with black skin have greater transepidermal water loss and decreased skin surface pH [50,51] and there is some evidence that more darkly pigmented skin has a more resistant barrier [52], but these results are inconclusive [53,54]. We also did not test skin pH during the study, an addition that would allow us to speak more to the link between low-pH solutions and irritation. Additionally, our study size was small for both practical and ethical reasons. Without established research on community handwashing and dermatitis we were not able to estimate sample size needs, and the IRB expressed that given this lack of evidence sample size should be limited for a preliminary study. Lastly, this study did not consider the efficacy of these handwashing methods for removal and inactivation of the Ebola virus, and future research is planned for early 2016 to investigate the efficacy of these six methods and the viability of the virus in wash water using human volunteers and bacteriophage surrogates for Ebola. Despite these limitations, we feel that our results are sufficient to inform community handwashing with these six methods and guide future research.

Our results suggest that the recommendation that chlorine should only be used for handwashing if there are no other options available is overly general and may not represent the best approach to limit increased disease transmission risk from development of dermatitis. We found that no handwashing method should be considered a significant transmission risk at frequencies of handwashing typical to communities. Further research should be done to explore the impact of these handwashing formulations in a context more similar in demographic makeup and environment to the outbreak area in West Africa, and should take into consideration the interaction of these methods with PPE and other potential irritants. Lastly, handwashing behavior change and compliance is a well-documented challenge [55]. Thus, while more research must be done before definitive conclusions about efficacy can be made, responders and communities concerned about Ebola should consider all available options and choose the most acceptable handwashing option that is readily available for sustained use.

## Supporting Information

S1 Dataset Handwashing Data. (XLSX)

S2 Dataset Data Dictionary. (XLSX)

S1 Checklist Consort Checklist. (DOC)

S1 Protocol Study Protocol. (DOC)
