# Supplementary material for: Seeking Clearer Recommendations for Hand Hygiene in Communities Facing Ebola: A Randomized Trial Investigating the Impact of Six Handwashing Methods on Skin Irritation and Dermatitis
Source: PLoS One. 2016 Dec 28;11(12):e0167378. doi: 10.1371/journal.pone.0167378 (PMC5193384; doi:10.1371/journal.pone.0167378)
Supplement: S1 Protocol — (DOC) [file pone.0167378.s004.doc]

# TUFTS MEDICAL CENTER/TUFTS UNIVERSITY RESEARCH PROTOCOL

**Version date:** 10-Aug-2015

**Principal Investigator:** Daniele Lantagne

**Study Title:** Comparison of soap, hand sanitizer, and 0.05% NaDCC, HTH, and NaOCl chlorine solutions in the development of dermatitis during frequent handwashing

1. Aim and Hypotheses

The aim of this study is to compare the impact of different methods of frequent hand washing used in healthcare settings on the development of irritant hand dermatitis. Subjects will be randomly assigned to one of six different handwashing methods – soap and water, hand sanitizer, 0.05% NaDCC chlorine solution, 0.05% HTH chlorine solution, and both stabilized and unstabilized 0.05% NaOCl chlorine solutions – and will wash their hands ten times daily using their assigned methods for four weeks while researchers monitor skin health and feel daily. We hypothesize that the development of dermatitis on the hands among subjects will vary depending on the substance used for handwashing.

1. **Background and Rationale**
2. **Background**:

Disinfection of hands and surfaces to protect both patients and healthcare providers from disease transmission is a top priority in health care settings worldwide, but frequent handwashing may also cause harm by excessively irritating skin. Health care providers strive to establish protocols that maintain safety from infection and also protect workers from the development of skin conditions such as dermatitis, but these recommendations are inconsistent and lack supporting evidence regarding the impact of washing on skin health. Many commonly used methods of hand washing and disinfection have been implicated in the development of irritant contact dermatitis (ICD)^1^, a condition of inflammation of the skin characterized by symptoms such as itching, redness, and skin swelling. These uncomfortable and occasionally painful symptoms of ICD are caused by skin barrier disruption, cytokine release, and cellular changes in the skin^2^. This disruption of the normally protective barrier of the skin can even make sufferers more susceptible to infection by presenting an entry point for disease agents, and especially severe cases may present with clear fissures in the hand that provide an obvious target for infection. ICD is distinct from allergic contact dermatitis (ACD). Although the clinical presentation of the two conditions are similar, ACD is a Type IV delayed hypersensitivity reaction and patients will display a consistent allergic reaction to the substance in question and will patch test positive for that substance^3^. By contrast, ICD is a non-specific response that may be experienced by a large percentage of a population regularly exposed to irritants commonly associated with dermatitis.

Soap and water, hand sanitizer, and chlorine solutions are all common handwashing solutions that have also been associated with concerns about dermatitis. The use of soap and water or hand sanitizer for handwashing is common in hospitals and clinics, but has been suggested as a common causes of ICD and is widely perceived among healthcare workers as an occupational hazard^1^. Chlorine solutions are also a common disinfectant used for both surfaces and hands, and due to their powerful disinfecting ability they are particularly widely used in emergency and high-risk scenarios such as the recent outbreak of Ebola Virus Disease (EVD) in West Africa. Chlorine solution can be made multiple ways using different compounds that provide the free chlorine that acts as a disinfectant. These compounds differ in their chemical properties and therefore may differ in the risk they pose to the integrity of the skin barrier on hands. (In Table 1 we provide a summary of the differences in basic properties and common additives among the solutions this study proposes to investigate.) There is some evidence that handwashing with chlorine solutions produced from sodium hypochlorite (NaOCl) and High Test Hypochlorite (HTH) may be linked to ICD^4–7^, however adequate attention has not been paid to the additives that often exist in these solutions that may act as stronger irritants than the active agents themselves^8^. For example, unstablized NaOCl has a neutral pH, while the stabilized solution has a much higher pH because of the additions of NaOH. This difference in pH is one factor that might have an impact on dermatitis development. This risk of ICD has not been demonstrated for chlorine solutions made from powdered sodium dichloroisocyanurate (NaDCC). The evidence for the risks associated with all of these handwashing solutions is inconsistent and predominantly observational, making it difficult to disentangle the impact of the handwashing methods themselves from the environments in which they are used.

However this evidence on the impact of these substances on skin relies heavily on observational studies in environments that contain many other irritants^1,4,9,10^, or on studies of accidental exposure to higher levels of substances than is recommended for handwashing. Studies focused on workers in health care and other occupational settings experiencing irritants within their usual context and are not adequately able to separate the effect of handwashing substances from the environment. Indeed studies have shown that there are numerous factors including weather and baseline reactivity to substances that impact dermatitis^1^. Other studies focus on the potential for these substances to induce an irritant reactions, but not within a concentration and protocol accepted for handwashing^3,5,6^. This study will examine the impact of these substances when used appropriately for handwashing in a context where other potential irritants are carefully controlled and tracked to provide high quality evidence on the direct impact of these substances.

|  | **NaDCC** | **HTH** | **NaOCl (Stabilized)** | **NaOCl (Unstabilized)** |
| --- | --- | --- | --- | --- |
| *Original Product Form* | Powder or Tablets | Powder or Tablets | Liquid | Liquid |
| *Chemical Structure* | 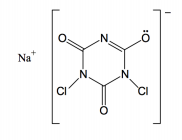 | 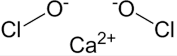 | 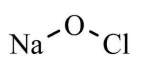 | 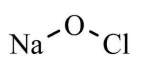 |
| *Added Compounds* | 98% Pure (No added compounds) | Compounds to: prevent powdering, prevent absorption of moisture, or help tablet dissolve | Stabilizers (NaOH), Surfactants, Sud suppresors | None |
| *pH* | 6-7 | 10.4 – 10.8 | 10.8-11.4 | ~7 |

*Table 1. Summary of Chlorine Solutions*

Because of this lack of evidence, the recommendations by international agencies and partnerships providing guidelines for safe hand washing and disinfection have been inconsistent and even contradictory. In standard hospital and clinic settings, both the WHO and the CDC recommend handwashing with soap and water or hand sanitizer use, but also acknowledge that frequent use of either method may result in disruption of the normal physiology of the skin barrier on hands^11,9^. In the context of the recent EVD outbreak in West Africa, international humanitarian organizations have focused on the use of chlorine for handwashing. Doctors Without Borders (MSF) recommends that buckets of 0.05% chlorine solution are prepared using powdered HTH or powdered NaDCC and placed around Ebola Treatment Centers (ETCs) for regular hand washing use^12^. These recommendations have spilled over into surrounding communities, with 0.05% chlorine solution adopted by government, health, and commercial facilities in West Africa requiring hand washing before entry^13^. An international organization was recently awarded 5 million euros to install chlorine-based handwashing stations throughout urban and rural areas of Guinea. The WHO recommendation contradicts these suggestions – they advocate hand washing with soap and water or with hand sanitizer containing humectants to prevent skin drying. Hand washing with chlorine, they say, “is not ideal because this may lead to skin lesions, which could increase risk of infection, and because prepared dilutions might be inaccurate.” However they do recommend that if other options are not available or feasible chlorine solution should be available for handwashing^14^.

Soap and water, hand sanitizer, and chlorine solutions using NaDCC, HTH, and NaOCl are all commonly used in both standard hospital settings and emergency outbreak setting for disinfection of hands to prevent transmission of infection, but the evidence about the risk that these substances may all pose for the development of ICD and subsequent higher risk of infection is inadequate to provide consistent recommendations to healthcare workers and emergency responders. This study will seek to provide clear evidence on this impact of handwashing using these methods by observing the impact of frequent handwashing in a controlled environment.

#### Rationale: There is a lack of evidence about the level of risk different handwashing methods pose for the disruption of the natural skin barrier on hands and development of irritant contact dermatitis. In both traditional healthcare settings and in disease outbreak settings there is a strong need for methods that both provide adequate hand disinfection and protect healthcare workers from the added risk that ICD may pose by disrupting skin’s natural protection. Healthcare workers and the dermatologists who treat occupationally acquired ICD are seeking clearer recommendations for regular handwashing and alternative suggestions for individuals displaying particular sensitivity to commonly used methods, and the international humanitarian community is asking for consistent evidence-based guidance on handwashing in the particularly risky situations where they face highly dangerous infectious diseases such as EVD.

It is difficult to distinguish the causes of dermatitis in these populations, since healthcare workers and all those who wash hands regularly are commonly exposed to a range of substances acting as irritants that may cause dermatitis^1^. Therefore there is a need to study the impact of these handwashing methods while controlling for other substances that may cause a skin reaction. The results of this study will provide evidence-based recommendations aligning contradictory guidance to healthcare workers in settings across the globe, and are particularly relevant to international organizations concerned with the most recent EVD outbreak. The outbreak in West Africa is the largest to date, establishing the potential for large and devastating outbreaks with reproductive rates >1^15^. Clear handwashing protocols are an essential practical recommendation to prevent future outbreaks attaining the size and duration of the most recent outbreak devastating West Africa.

1. **Research Plan**

### Experimental design:

### This study consists of an individually randomized controlled trial with six arms to assess the impact of six different methods of frequent handwashing (soap and water, hand sanitizer, 0.05% chlorine solution made with NaDCC, 0.05% chlorine solution made with HTH, 0.05% stabilized chlorine solution made with NaOCl, 0.05% unstabilized chlorine solution made with NaOCl) on development of dermatitis.

*Recruitment and Enrollment*: Subjects will be recruited using flyers posted around the Medford/Somerville area and newspaper advertisements taken out in the Tufts Daily, the Somerville Journal and the Medford Journal (Appendix A). These advertisements will describe the purpose and requirements of the study and provide contact information for the study team. The flyer will also be distributed as an image to public listservs and social media groups such as the Medford Moms email list and Tufts campus email lists. Individuals who feel that they meet the study’s criteria may attend one of several 1 hour meetings during which researchers will explain the study in detail (Frequently Asked Questions in Appendix B). Potential volunteers will be given the opportunity to ask questions about the study in a group setting. After the meeting subjects will meet with researchers individually to ask further questions they may be uncomfortable asking in a public setting and study staff will collect written informed consent (Appendix C) in a private setting. Subjects who consent to the study will then be asked to schedule a time to complete the baseline survey and data collection. Potential volunteers who do not wish to attend a group meeting or are unable to attend at the posted time may schedule a meeting with researchers to learn about the study individually and provide informed consent if they choose to participate.

*Baseline Assessment and Exclusion Criteria*: At baseline, subjects will 1) respond to a questionnaire (Appendix D), 2) have to a hand exam (Appendix E) and 3) have a patch test applied to test for allergy to any of the study substances (Appendix F). This baseline data will also be used to confirm that subjects do not meet any of the study’s exclusion criteria. The questionnaire will include demographic and contact information, information about personal history of dermatitis and atopic disposition (history of seasonal allergies, asthma, childhood eczema, and hay fever), and typical handwashing behavior. The hand exam will look for signs of current dermatitis or baseline skin abnormalities and assess a score from 0-360 using the Hand Eczema Severity Index (HECSI)^16^. The patch testing kit (8mm Finn Chambers® on Scanpor® Tape) will be applied using dilutions of all six substances included in the study (soap, hand sanitizer, NaDCC, HTH, stabilized NaOCl, unstabilized NaOCl) in two locations - on the upper back to comply with the gold standard of testing and on the arm to allow for ease of viewing and reporting by subjects.

Subjects will be instructed on how to remove the patches and mark the area tested with a surgical marker 48 hours after application. Subjects will take photos of the patch site and provide the photos to researchers via email, and will also be instructed to notify the study team if they observe any redness inside the target areas. Subjects will also be given the option to report in person to have a photo of the area taken if they are unable to take a photo or uncomfortable emailing the photo to researchers. Subjects will repeat the observation and provide another photo on the 96-hour mark. On Day 7 after application subjects will be seen in person by research staff, who will verify negative patch testing and refer any suspected positive cases to Dr. Pamela Scheinman, co-PI on the study, for verification.

Subjects who are pregnant or trying to become pregnant and those with a history of dermatitis, any baseline skin abnormalities upon exam, a positive patch test to any study substance, or who are employed in a profession in which hands are regularly exposed to irritants (e.g. healthcare workers, mechanics, hairdressers/cosmetologists, bartenders or dishwashers) and those with a self-reported history of mental health issues that may be triggered by a study related to hygiene will be excluded from the study.

*Randomization*: Subjects will be randomized into one of the six treatment groups using a computer-generated randomization list prepared by a researcher not involved in the running of the trial.


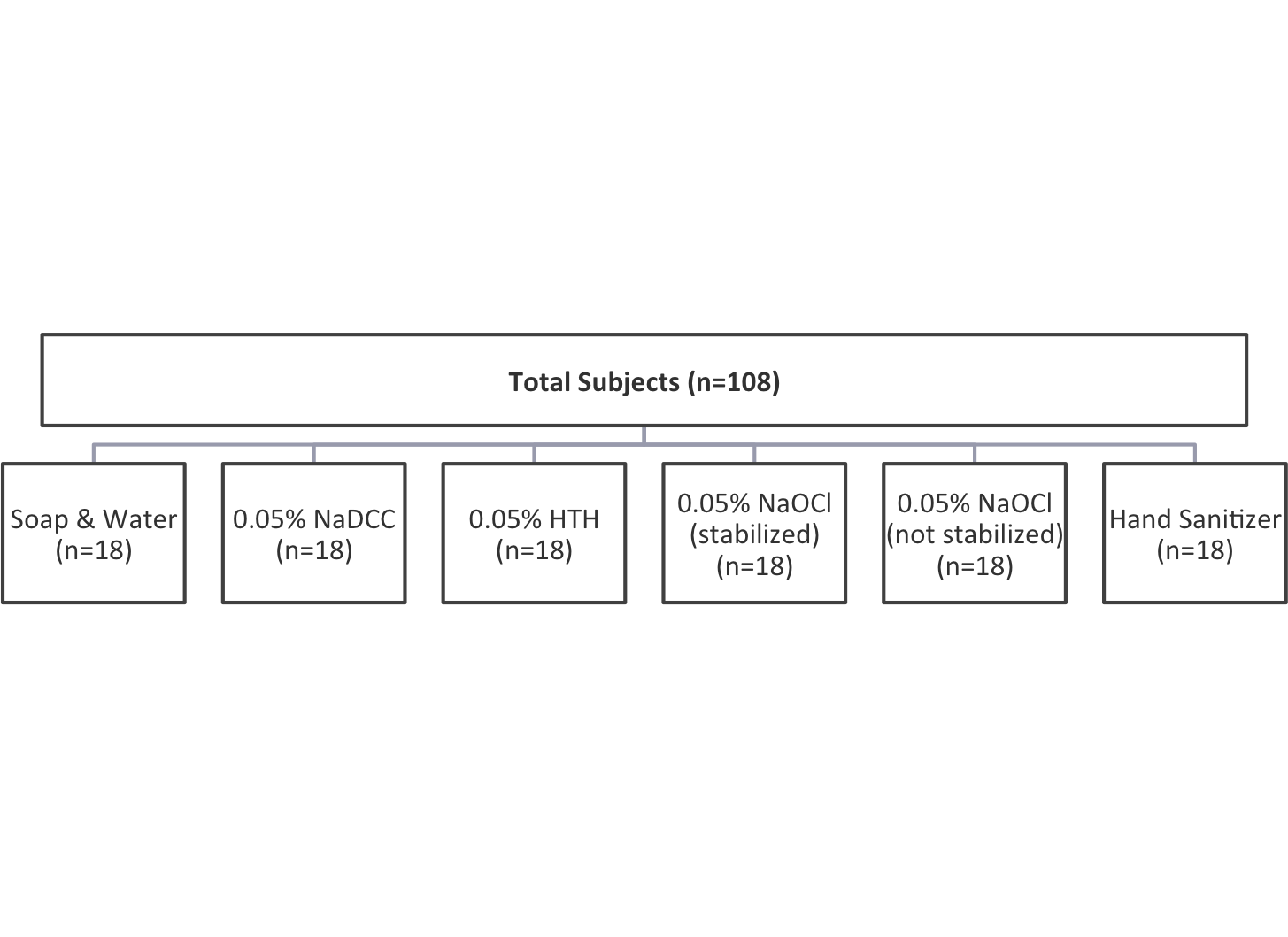


*Fig 1. Treatment Groups*

*Intervention*: At the beginning of the intervention subjects will be given a drawstring bag containing handwashing materials and hypoallergenic hygiene items including shampoo, conditioner, soap, and fragrance-free Vaseline along with instructions for use (Appendix G). Subjects will be asked to use the provided hygiene items in lieu of their usual products in order to control for possible other causes of skin irritation for the duration of the experiment. Subjects will be told that they can moisturize using the Vaseline as much as they would like. Vaseline is the best choice for this purpose because it is hypoallergenic, consistent with the way that healthcare workers commonly manage any skin itchiness and dryness, and consistent with available options for moisturizing in both the United States and West Africa. During the four weeks of intervention, subjects will be asked to wash their hands 10 times daily with the substance assigned to their treatment group, and instructed to use the soap provided in the hygiene kit for any hand or body washing they may do above and beyond the handwashing with their assigned substance (instructions in Appendix H). Each evening subjects will be asked to report to Anderson Hall on the Tufts Medford campus to pick up a refill of their assigned handwashing materials. Refills of the hygiene items will be available as needed. During the pickup time each day, subjects will also answer a brief questionnaire administered on tablets assessing adherence to the handwashing protocol, self-assessment of hand feel (including itchiness, pain, redness, flaking, cracks, skin thickening or swelling), receive a brief physical hand examination from study personnel, and take photos of both sides of each hand (Appendix I). The photos and hand exam scores will be reviewed daily for signs of dermatitis or skin abnormalities, and any suspected cases will be referred to Dr. Scheinman for treatment and assessment to determine whether continuation in the study is recommended. Dermatitis will be assessed using the HECSI scale, and the severity of the score will be used to guide the most likely course of action as described in Table 2 below.

*Endline*: Endline data collection will occur on the last day of the intervention. All subjects will respond to the daily questionnaire and receive a hand exam as usual, and patch testing will be applied a second time according to the same protocol used during baseline. As during the previous assessment, subjects will remove the patch, mark the area, and take pictures after 48 and 96 hours and the area will be examined in person by a researcher on Day 7. Any suspected positive cases will be referred to Dr. Scheinman for verification and subjects with a confirmed positive will be recommended to avoid the substance in the future.

*Data Analysis*: As a preliminary study, the analysis for this study will be descriptive and foundational for further studies examining substances that show promise. The primary outcome of this study is the development of dermatitis, as measured by both HECSI score and self-perception index where subjects are asked to describe symptoms and discomfort experienced on a scale from 1-10. The HECSI score is a highly sensitive tool designed for use in research and epidemiological studies, and will serve as the gold standard of measurement for clinical dermatitis. However perception and discomfort are highly important aspects of the impact of dermatitis on patients, meaning the self-perception index adds an important dimension to the measurement of dermatitis as an outcome. We plan to compare the proportion of subjects presenting with signs and symptoms of dermatitis in each treatment group, and to stratify the results by gender and history of atopy in order to control for potential confounders.


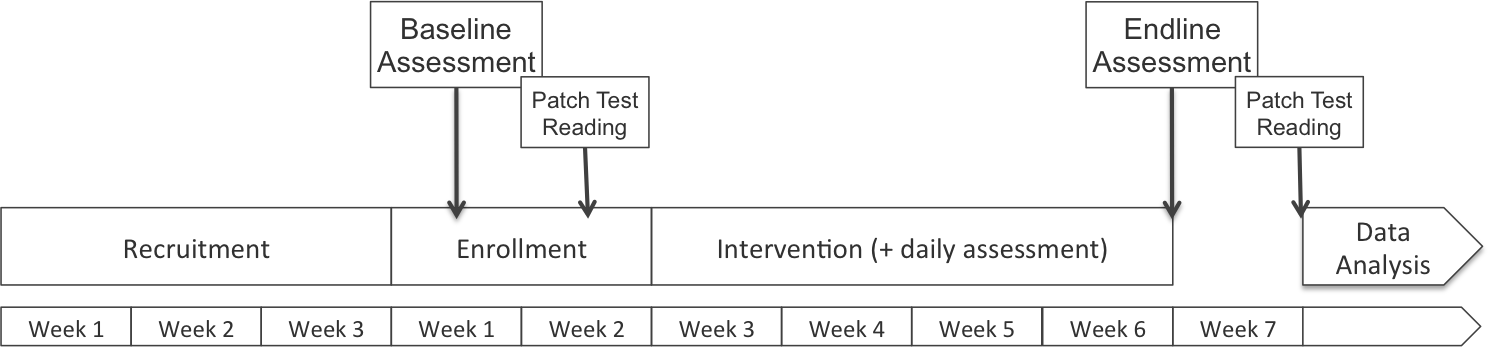


*Figure 2. Study Timeline*

### Sample size and statistical analysis(es) :

### We plan to enroll 108 total subjects in the study, with 18 randomized to each arm. We will target this number with the understanding that minor attrition may occur, and estimate that we need 15 subjects per arm to complete the study to achieve our goals.

**Randomization Methods**: Randomization will be done after patch-testing results are read, to ensure that no subjects meeting exclusion criteria are involved in the study intervention. A researcher who does not interact directly with subjects will perform randomization. Subjects will be numbered (1-108). 18 subjects will be assigned to each group. A random number generated will be used to generate 18 numbers between 1 and 108. These subjects will be assigned to the first intervention group. Random number generation will be repeated in this way five times, with subjects corresponding to the number assigned to the treatment group for the round. The 18 remaining subjects will be assigned to the last treatment group.

**Blinding**: Researchers assessing HECSI scores for the hand exam and applying and interpreting results of the patch testing will be blinded to subject treatment group. Daily surveys will not record treatment arm and subjects will be instructed not to identify their group to examiners.

It is not possible to blind subjects to treatment, because the difference between soap and water, hand sanitizer, and chlorine solutions are obvious and cannot be obscured. However subjects assigned to one of the chlorine solution treatment groups will not be told which solution they have been assigned.

**Sample Size Calculations:** Because there are no previous handwashing studies using chlorine, soap, or hand sanitizer for handwashing that can provide information on the number of subjects likely to develop dermatitis we are unable to calculate a meaningful sample size for statistical significance. We feel that this small sample size is appropriate for a first study. For example if 20-100% of participants in a given arm develop dermatitis that will suggest that the method is problematic and should not be recommended, but if 0% of participants develop dermatitis this will be good evidence that the method has potential and should be studied further. This study is intended to be a first investigation that can provide information that could be used in the future for sample size calculations for a larger trial if any of the handwashing materials show potential.

### Subject Characteristics

- 1. *Subject criteria*: Subjects will be men and women without existing skin conditions between the age of 18 and 65.

1. *Inclusion criteria*: All subjects should be English-speaking adults between the ages of 18-65. Because this is a not for benefit study and the documents will not be translated, the study will be restricted to English-speakers. This will not affect the scientific validity of the study because language is not a characteristic expected to relate to the health of skin on hands.
2. *Exclusion criteria*: Exclusion criteria will be assessed at enrollment using the baseline survey, a hand exam, and patch testing for all substances included in the study. Subjects indicating a history of dermatitis, who are currently pregnant or trying to become pregnant, who patch test for any substance in the trial, who display baseline skin abnormalities (e.g. open sores or dermatitis) during a hand exam, or who are employed in a profession in which hands are regularly exposed to irritants (for example healthcare workers, mechanics, hairdressers/cosmetologists, bartenders or dishwashers) will be excluded from the sample.
3. *Withdrawal/Termination criteria*: Subjects are free to withdraw from the study at any time without penalty. Any subject presenting with moderate to severe dermatitis (assessed score > 270 on the HECSI scale or as diagnosed by Dr. Scheinman) will be removed from the study unless Dr. Scheinman determines that the reaction observed is not clinically significant. All subjects will receive prompt medical attention if necessary and will subsequently be cleared for continuing participation by Dr. Scheinman or removed from the study. Subjects must participate in 95% of study activities (daily questionnaire and hand exam, daily pickup of new materials, 10x handwashing daily) in order to continue in the study.
4. Subjects may not participate in other studies while participating in this study

### Risk/benefit assessment: We expect that the activities in this study place subjects at a minimal level of risk, which is consistent across the treatment arms, as the intervention activities are not outside the range of normal hand hygiene activity and do not present a risk great than that encountered in daily life.

1. Physical risk: Mild risk of development of dermatitis, no greater than the risk of frequent (but within normal) handwashing in daily life or work.
2. Psychological risk: None expected (Subjects who self identify as having mental health issues related to hygiene activities will be excluded from the study)
3. Social risk: Subjects may find that the time required to pick up new handwashing materials each day poses a burden
4. Economic risk: None expected
5. Benefit of participating in the study: We don’t expect participants to experience a direct benefit from this study. However the results of this study are expected to benefit anyone, healthcare workers and otherwise, who wash their hands frequently by providing evidence of the methods least likely to cause problems for hand health. More broadly, we expect the health care and humanitarian community to benefit from the recommendations resulting from this and subsequent studies that will provide evidence about which methods are most likely to provide adequate disinfection while also keeping the natural protection of the skin barrier intact.

### Specific methods and techniques used throughout the study: Data will be collected using surveys to assess subject history at baseline, survey to collect information on compliance and hand perception and hand exams throughout the intervention, and patch testing at baseline and endline assessments. All methods are non-therapeutic and carried out for research purposes only.

### Laboratory tests: None

### Study Procedures:

### *Hand exam*: Subjects will receive a visual inspection of hands assessing a score for the severity of erythema, infiltration/papulation, vesicles, fissures, scaling, and edema at different locations on the hands and the extent of the area affected. This evaluation is used to calculate a HECSI score at baseline, and daily during the intervention using the form in Appendix E. Photos of hands will also be taken daily.

*Patch Testing*: Subjects will have patch testing done at baseline and endline for all the substances included in the study with data and results recorded in the form shown in Appendix F. Each substance used in the study will be diluted and placed on the skin under a patch sealed with hypoallergenic tape (8mm Finn Chambers® on Scanpor® Tape) in two areas – on the back and upper arm. Testing on the back provides the gold standard, and patches on the arm will allow for easy observation and reporting by subjects. 48 hours after application, subjects will be asked to remove the patch, identify the testing area by marking it with a surgical marker, and take a photo of the area to provide to researchers. Subjects will provide another photo 96 hours after application. Subjects will be instructed to contact study staff if they observe any redness or suspect a reaction within one of the six testing areas. 7 days after testing, researchers will visually inspect the patch-testing site. Suspected positives will be referred to Dr. Scheinman for confirmation.

### Subject Timeline: Subjects will be enrolled during a four week recruitment period during which informed consent will be collected. Subjects will be enrolled in the study for 7 weeks total, 4 of which will consist of daily intervention.

*Week 1*: Subjects will respond to the baseline survey, receive a hand exam, and have the baseline patch testing kit applied. After 48 hours, they will remove the patch and take a photo of the area. Subjects will take another photo after 96 hours and will be instructed to report any redness, bumps, or scaling at the patch site.

*Week 2*: On day 7 after patch testing subjects will receive an in-person examination of the patch test area. Any subjects meeting the exclusion criteria on the basis of the baseline survey, hand exam, or patch testing data will be excluded from the sample. After any subjects meeting exclusion criteria are removed from the sample, subjects will be randomized into one of the six treatment groups.

*Week 3 (Intervention Week 1)*: Week 3 is the first week of the intervention. Subjects will receive an orientation in handwashing procedures for their assigned treatment group and will wash their hands 10x daily with the substance assigned to their treatment group. Subjects will report daily to pick up handwashing materials for the next day and answer a short survey about compliance and hand feel, including a hand exam and photos of hands.

*Week 4 (Intervention Week 2)*: Week 4 is the second week of the intervention. Subjects will wash their hands 10x daily with the substance assigned to their treatment group, and will report daily to pick up handwashing materials for the next day and answer a short survey about compliance and hand feel, including a hand exam and photos of hands.

*Week 5 (Intervention Week 3)*: Week 5 is the third week of the intervention. Subjects will wash their hands 10x daily with the substance assigned to their treatment group, and will report daily to pick up handwashing materials for the next day and answer a short survey about compliance and hand feel, including a hand exam and photos of hands.

*Week 6 (Intervention Week 4)*: Week 5 is the fourth and final week of the intervention. Subjects will wash their hands 10x daily with the substance assigned to their treatment group, and will report daily to pick up handwashing materials for the next day and answer a short survey about compliance and hand feel, including a hand exam and photos of hands. At the end of Week 6 subjects will be given the endline assessment, which includes a second round of patch testing.

*Week 7*: At the end of Week 7 subjects will have the results of endline patch testing read on Day 7 after application, concluding their involvement in the study.


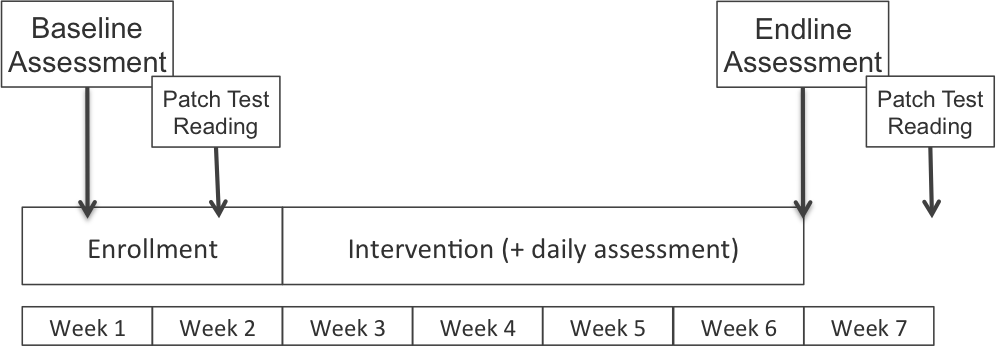


*Fig 2. Subject Timeline*

1. **Assessment of Subject Safety and Development of a Data and Safety Monitoring Plan**
   - - 1. **Definition of Serious Adverse Event (SAE) and Adverse Event (AE) for this study**:

Serious Adverse Events and Adverse Events will be defined as stated in the Tufts Medical Center/Tufts University Health Sciences IRB’s Unanticipated Problem and Adverse Event Reporting Policy. An adverse event is “any untoward or unfavorable medical occurrence in a human subject, including any abnormal physical exam or laboratory finding, symptom, or disease, temporally associated with a subject’s participation in the research.” Subjects will be seen daily by researchers and receive a hand exam, allowing any problems encountered to be identified immediately. Dr. Scheinman will examine subjects experiencing any adverse symptoms or discomfort and is responsible for determining the severity of the issue and whether the issue is likely related to the research activities. Table 2 provides a framework for response to suspected cases of dermatitis. These ranges and treatment options are guidelines, and actions to be taken are ultimately at the discretion of Dr. Scheinman. Subjects who have a very low level of discomfort may be assessed but not need any treatment. Subjects with more significant symptoms or discomfort may be provided with over the counter treatment or prescription treatment based on Dr. Scheinman’s assessment. If subjects experience a HECSI score above 270 (out of 360) they will most likely be removed from the study. Subjects are also free to withdraw at any time and Dr. Scheinman may remove subjects at her discretion.

| **Level** | **HECSI Score** | **Consult** | **Treatment** | **Study Implications** |
| --- | --- | --- | --- | --- |
| 1 | 68-134 | Telephone Consult | None | None |
| 2 | 135-202 | Telephone Consult | Over the counter | None |
| 3 | 203-269 | In-person Consult | Prescription | None |
| 4 | 270-360 | In-person Consult | Prescription | Participation terminated |

*Table 2. Response to Suspected Dermatitis*

A Serious Adverse Event (SAE) is defined by the Tufts IRB as: “any AE that: 1) Results in death, or 2) Is life-threatening, or 3) Results in hospitalization or prolongation of existing hospitalization, or 4) Results in a persistent or significant disability/incapacitation, or 5) Results in a congenital anomaly/birth defect, or 6) May jeopardize the subject’s health and may require medical or surgical intervention to prevent one of the other outcomes listed above.” Dr. Scheinman is responsible for determining the severity of the issue and whether the issue is likely related to the research activities. SAEs for this study are unanticipated, and will be treated as an Unanticipated Problem if Dr. Scheinman determines that they may be related to study activities.

This study will not have a Data and Safety Monitoring Board because it is a single site study with minimal risk.

- - - 1. **Reporting timeframe for SAEs and AEs**:

Any AEs that are non-serious but clinically significant will be summarized using the Tufts Summary Report Form and submitted at the end of the study, or at the point of a continuing review if necessary. Unanticipated problems will be reported to the IRB within 5 days if Dr. Scheinman determines that the event is likely related to study participation. If the event appears to be unrelated to study participation, the SAE report will be submitted within 15 business days. Dr. Scheinman is responsible for assessing the severity and relationship to study activities of any adverse events, which will determine which type of reporting is required. Reports and summaries will not contain identifying information about subjects involved.

- - - 1. **Accountability procedures as they relate to drugs, devices, and data:**

*Intervention Materials:*

Chlorine solutions will be made fresh regularly and concentrations will be confirmed using the Hach 8209 sodium thiosulfate digital titration kit. Soap and hand sanitizer will be used within the product expiration dates.

*Data Collection*

Data collection forms will be reviewed on a daily basis for completeness and to quickly identify major errors. Dr. Scheinman will review HECSI scores and photos of hands to identify any subjects that may be in need of follow up. Compliance with the study intervention will be measured by self-report in the daily questionnaire and by observation of the amount of handwashing substance left by each subject on a daily basis.

## Subject Participation

- 1. **Recruitment**: Subjects will be recruited using posters, newspaper advertisements, and email/social media messages which will contain a brief description of the study purpose, dates, and eligibility criteria to be circulated both physically and via email and with directions to respond to a website form with interest. The posters will be distributed throughout the Medford/Somerville area, ads will be placed in the Tufts Daily, Medford Journal, and Somerville Journal, and email and social media messages will be distributed publicly to listservs and groups for adults located in the Medford/Somerville area. The recruitment poster is included in Appendix X and newspaper advertisement in Appendix X. Social media and email advertisements will simply consist of an image of the poster.
  2. **Registration**: No subject registration is required.
  3. **Screening Interview/questionnaire**: Screening questions will be included in the baseline survey (Appendix D), administered after subjects have consented to participation in the study. Research staff (Tufts graduate and undergraduate students trained in survey administration) will administer the survey to subjects in person on Tufts Medford campus.
  4. **Transportation**: Subjects will be responsible for their own transportation to pick up handwashing materials daily and attend baseline and endline assessments on Tufts Medford campus. In case any subject develops dermatitis or skin abnormalities requiring treatment in person, subjects will be reimbursed up to $100 for transportation to receive a consultation with Dr. Scheinman.
  5. **Informed consent process and timing of obtaining of consent**: Research staff will describe the details of the study purpose, requirements, and inclusion and exclusion criteria to subjects in a group meeting, during which potential participants will have the opportunity to ask questions.

After the meeting, potential participants will meet with research staff one on one in a private setting to review the details of the consent form and to provide the opportunity to ask questions that they felt uncomfortable asking in a public setting. Research staff will then collect written consent if the subject indicates that they are willing to participate. Staff will be responsible for assessing the subject for signs of cognitive or decisional impairment, and will not allow anyone to consent who appears unable to provide informed consent. Subjects will be given as much time as they need to consider whether or not they would like to enroll, and may return with the signed consent form anytime before the end of the scheduling window for the baseline survey.

- - 1. **If non-English speaking persons will be enrolled, state the informed consent process for enrolling the subjects, including who will conduct the consent interview, use of interpreters, translated documents, etc.:** Because this is a not for benefit study and the documents will not be translated, non-English speaking subjects will not be enrolled in this study.

**(NOTE: Exclusion of non-English speaking subjects from research requires ethical and scientific justification. This justification must be stated elsewhere in the protocol.)**

1. **Location where study will be performed**: All study procedures will take place on the Tufts Medford campus. Consent forms will be stored in a locked cabinet on campus, and all study data will be collected electronically and stored on a secure, password-protected server.
2. **Personnel who will conduct the study, including**:
   - 1. **Present during study procedure(s) and their proximity during the study**:

Daniele Lantagne (co-PI) will be present during the baseline assessment, and will be working from the Tufts Medford campus for the duration of the study.

Pamela Scheinman (co-PI) will be present during some administration of hand exams and patch testing and during review of Day 7 patch testing results for the baseline and endline assessment, and will be based at her clinic at Brigham and Women’s Hospital for the duration of the study.

Marlene Wolfe (Research Coordinator, Tufts PhD student) will be present during consent, baseline, and endline assessments, and daily administration of handwashing materials and surveys during the intervention along with Emma Wells (Tufts Undergraduate Student) as an assistant. Marlene will be working from the Tufts Medford campus for the duration of the study.

1. **Primary responsibility for the following activities**:
   - 1. **Obtaining informed consent**: Marlene Wolfe (Research Coordinator, Tufts PhD student) will be responsible for obtaining informed consent and will be assisted by other Tufts graduate and undergraduate students.
     2. **Providing on-going information to the study sponsor and the IRB**: Daniele Lantagne will be responsible for providing ongoing information to the study sponsor (USAID/OFDA) and the IRB and will be assisted by the research coordinator, Marlene Wolfe.
     3. **Maintaining participant's research records**: Marlene Wolfe (Research Coordinator, Tufts PhD student) will be responsible for maintaining security and confidentiality of participant’s research records.
2. **Subject fees**: There are no fees associated with participation in this study. Travel costs associated with normal study activities will not be reimbursed, however in the case of an adverse event, transportation for medical consultation with Dr. Scheinman.
3. **Study results**: Study results will not be disseminated directly to subjects, however subjects will be asked if they would like to be notified when results are published.
4. **Procedures to protect subject confidentiality**: Although this study presents a low risk of breach of confidentiality, procedures will be put in place to protect participant identity and associated information. Data is collected for research purposes only. Data (including photos of subject’s hands) will be password-protected and anonymized for analysis and identifying characteristics recorded will be minimized and used only for logistical purpose during the intervention stage of the study. Information will not be given without a subject’s consent. Photos will be used internally to assess hand health and confirm accuracy of data and will not be shared without permission from subjects. Photos will be stored for one year after the end of the intervention period and then destroyed. Any Adverse Event reports will include only subject ID numbers without any identifying information.
5. **Confidentiality**:
   - - 1. **Certificate of Confidentiality**: N/A
       2. **How data will be coded, recorded, and stored to protect confidentiality**: Each individual enrolled in the study will be given a unique identifying number, and data on identifying characteristics will be stored separately from other study data. Data will be recorded electronically using surveys administered on tablets, and dataset will be stored on a secure, password-protected server.
       3. **Parties who will have access to the data, including the key to the identity code**: The PIs and Research Coordinator will have access to the data and the key to the identity code.
       4. **Parties who will have access to research records:** The PIs and Research Coordinator will have access to research records.
6. **Collaboration**: This study is part of a grant from USAID/OFDA funding five separate investigations (#1-#5) relevant to disinfection to prevent Ebola transmission. Only one of the five investigations (the handwashing study described herein, which is #3) requires IRB approval, the remaining four projects (#1, #2, #4, and #5) are laboratory-based investigations. The University of Brighton and Brigham and Women’s Hospital are named along with Tufts University on all study branded materials for each of the five investigations in accordance with the Branding and Marketing Strategy developed with and required by USAID (attached). The University of Brighton is leading two of the laboratory-based investigations (#4 and #5) in the United Kingom, and Tufts University is leading the remaining three (#1, #2, #3) on the Tufts Medford Campus. Brigham and Women’s Hospital is subcontracted for the handwashing investigation only (#3), as Dr. Scheinman will provide clinical expertise as a co-PI from this institution. The full grant application provided includes a description of all investigations (#1-#5).
7. **Alternatives**: Alternatives are not applicable, as study is not enrolling subjects seeking treatment for an existing condition. The alternative to participating in this study is to not participate.
8. **How new information will be conveyed to the study subject and how it will be documented**:

Contact information including phone number and email address will be collected for each patient, and patients will be notified of any changes in the study in writing either through email or in person. These written records of changes will be archived and a description of changes included in any write up of study methods.

1. **Payment, including a prorated plan for payment**: Subjects will receive a total of $600 after successful completion of all study activities. Subjects will receive $100 for each round of patch testing is completed, and $100 for each week of the intervention completed. Although this study presents minimal risk to subjects, a high level of compensation is called for due to the study’s labor-intensive nature including consistent attention to details and the high level of compliance to intervention activities and attendance at assessments over the course of 7 weeks and the inconvenience of patch testing procedures. The fees will be paid in three stages: $100 after the baseline assessment, $200 at the midpoint of the intervention, and $300 after the endline assessment.
2. **Payment for a research-related injury**: Dr. Scheinman will examine any subjects showing signs of dermatitis. Over the counter treatment will be provided by the research team if necessary, but subjects will be covered by personal medical insurance for consultations at Dr. Scheinman’s clinic. If subjects do not have sufficient insurance the study will cover the costs of this consultation. Subjects will be reimbursed up to $100 for transportation to a consultation at Dr. Scheinman’s office. Subjects will not be financially compensated for any research-related injuries.
3. **Outcome**: We expect low levels of our primary outcome of dermatitis (as measured by visual dermatitis scale assessed by study staff) and secondary outcome of discomfort (as measured by self-perception of hand feel) and any level of dermatitis, including 0%, will provide valuable data. However a high level of compliance is necessary for the success of the study and will be measured by self-report and observation of materials. We define high compliance as >95% completion of study intervention activities, as measured by survey completion, pickup of materials, and self reported handwashing 10x per day.

1. **Tissue banking considerations**: None

# VULNERABLE POPULATIONS: This study will not involve any vulnerable populations.

**REFERENCES**

1. Callahan A, Baron E, Fekedulegn D, et al. Winter season, frequent hand washing, and irritant patch test reactions to detergents are associated with hand dermatitis in healthcare workers. *Dermatitis*. 2013;24(4):170-175. doi:10.1016/j.jsbmb.2011.07.002.Identification.

2. Staff MC. Diseases and Conditions - Dermatitis. 2015. http://www.mayoclinic.org/diseases-conditions/dermatitis-eczema/basics/definition/con-20032183.

3. Hostynek JJ, Patrick E, Younger B, Maibach HI. Hypochlorite sensitivity in man. *Contact Dermatitis*. 1989;20(1):32-37.

4. Hansen KS. Occupational dermatoses in hospital cleaning women. *Contact Dermatitis*. 1983;9(5):343-351.

5. Eun HC, Lee AY, Lee YS. Sodium hypochlorite dermatitis. *Contact Dermatitis*. 1984;11(1).

6. Habets JMW, Geursen-Reitsma AM, Stolz E, Joost T van. Sensitization to sodium hypochlorite causing hand dermatitis. *Contact Dermatitis*. 1986;15(3):140-142.

7. Osmundsen PE. Contact dermatitis due to sodium hypochlorite. *Contact Dermatitis*. 1978;4(3):177-178.

8. Hostynek JJ, Wilhelm KP, Cua AB, Maibach HI. Irritation factors of sodium hypochlorite solutions in human skin. *Contact Dermatitis*. 1990;23(5):316-324. doi:10.1111/j.1600-0536.1990.tb05165.x.

9. Boyce JM, Pittet D. *Guideline for Hand Hygiene in Health-Care Settings. Recommendations of the Healthcare Infection Control Practices Advisory Committee and the HICPAC/SHEA/APIC/IDSA Hand Hygiene Task Force. Society for Healthcare Epidemiology of America/Association for Prof*.; 2002. doi:10.1086/503164.

10. Slodownik D, Lee A, Nixon R. Irritant contact dermatitis: A review. *Australas J Dermatol*. 2008;49(1):1-11. doi:10.1111/j.1440-0960.2007.00409.x.

11. Organization WH. *WHO Guidlines on Hand Hygiene in Health Care, First Global Patient Safety Challenge, Clean Care Is Safer Care*.; 2009. http://whqlibdoc.who.int/publications/2009/9789241597906_eng.pdf.

12. Sterk E. *Médecins Sans Frontières - Filovirus Haemorrhagic Fever Guideline*.; 2008.

13. Emmanuel J, Nodye B. *Report to WHO: Assessment and Recommendations Regarding Management of Ebola-Contaminated Waste*.; 2015. https://noharm-global.org/sites/default/files/documents-files/3127/Report to WHO WASH and Geneva on Ebola final.pdf.

14. *WHO Report: Ebola Virus Disease ( EVD ) Key Questions and Answers Concerning Water , Sanitation and Hygiene*.; 2014.

15. *WHO Ebola Situation Report*.; 2015.

16. Held E, Skoet R, Johansen JD, Agner T. The hand eczema severity index (HECSI): A scoring system for clinical assessment of hand eczema. A study of inter- and intraobserver reliability. *Br J Dermatol*. 2005;152(2):302-307. doi:10.1111/j.1365-2133.2004.06305.x.
